# Hha has a defined regulatory role that is not dependent upon H-NS or StpA

**DOI:** 10.3389/fmicb.2015.00773

**Published:** 2015-07-30

**Authors:** Carla Solórzano, Shabarinath Srikumar, Rocío Canals, Antonio Juárez, Sonia Paytubi, Cristina Madrid

**Affiliations:** ^1^Departament de Microbiologia, Universitat de BarcelonaBarcelona, Spain; ^2^Institute of Integrative Biology, University of LiverpoolLiverpool, UK; ^3^Institut de Bioenginyeria de Catalunya, Parc Científic de BarcelonaBarcelona, Spain

**Keywords:** Hha, H-NS, StpA, *Salmonella*, motility, pathogenicity island, gene regulation

## Abstract

The Hha family of proteins is involved in the regulation of gene expression in enterobacteria by forming complexes with H-NS-like proteins. Whereas several amino acid residues of both proteins participate in the interaction, some of them play a key role. Residue D48 of Hha protein is essential for the interaction with H-NS, thus the D48N substitution in Hha protein abrogates H-NS/Hha interaction. Despite being a paralog of H-NS protein, StpA interacts with HhaD48N with higher affinity than with the wild type Hha protein. To analyze whether Hha is capable of acting independently of H-NS and StpA, we conducted transcriptomic analysis on the *hha* and *stpA* deletion strains and the *hha*D48N substitution strain of *Salmonella* Typhimurium using a custom microarray. The results obtained allowed the identification of 120 genes regulated by Hha in an H-NS/StpA-independent manner, 38% of which are horizontally acquired genes. A significant number of the identified genes are involved in functions related to cell motility, iron uptake, and pathogenicity. Thus, motility assays, siderophore detection and intra-macrophage replication assays were performed to confirm the transcriptomic data. Our findings point out the importance of Hha protein as an independent regulator in *S.* Typhimurium, highlighting a regulatory role on virulence.

## Introduction

One of the relevant features of bacterial cells is their ability to sense and adapt to a usually rapidly changing environment. Bacteria have developed several mechanisms to detect and transduce external stimuli resulting in modifications of the gene expression pattern. Nucleoid-associated proteins play relevant roles in bacteria, both organizing the chromosome and influencing gene expression. A well-known example is the nucleoid-associated protein H-NS. The H-NS protein is widely distributed within Gram-negative bacteria and is one of the best characterized examples of a modulator that influences gene expression in response to environmental stimuli (Dorman, 2007). In *Escherichia coli*, up to 5% of the genes are subjected to H-NS regulation (Hommais et al., 2001). In *Salmonella* Typhimurium, approximately 9% of the genes show an H-NS-dependent regulation (Lucchini et al., 2006; Navarre et al., 2006). Moreover, a significant 77% of temperature-dependent genes described in *S.* Typhimurium are modulated by H-NS (Ono et al., 2005). The H-NS protein consists of an N-terminal dimerization domain separated from a C-terminal DNA-binding domain by a linker region (Tendeng and Bertin, 2003). H-NS binding sites typically show curvature given by A-T rich sequences, a common trait found at promoters (Fang and Rimsky, 2008). The H-NS protein is not only capable of interacting with DNA but also with itself and other proteins. One of the best known examples of H-NS interacting protein is its paralog, StpA, that can form homomeric or heteromeric complexes *in vivo* mediated by the N-terminal domains of the proteins (Williams et al., 1996; Cusick and Belfort, 1998; Free et al., 1998; Johansson and Uhlin, 1999). Oligomerization of H-NS, forming extended protein filaments along target sequences, is critical for the regulatory role of the protein (Spurio et al., 1997; Dame et al., 2000; Badaut et al., 2002; Stella et al., 2005).

The Hha family of nucleoid associated proteins includes a group of sequence related low-molecular mass proteins. They are uniquely encoded by members of the Enterobacteriaceae (Madrid et al., 2007b) and are involved in modulation of virulence gene expression in response to environmental cues (Madrid et al., 2007a). Good examples of that are the regulation of α-haemolysin and the *esp* operons in *E. coli* (Nieto et al., 1991; Sharma and Zuerner, 2004; Sharma et al., 2005), and the modulation of *hilA* and SPI-2 virulence genes in *S*. Typhimurium (Fahlen et al., 2001; Coombes et al., 2005; Silphaduang et al., 2007; Vivero et al., 2008; Queiroz et al., 2011; Fàbrega and Vila, 2013). Genes coding for such proteins are present in one or more copies per chromosome or in transmissible elements such as conjugative plasmids (Madrid et al., 2007a). In addition to *hha*, the genome of *Salmonella* contains the *ydgT* gene, which encodes an Hha paralog (Paytubi et al., 2004).

Hha interacts with H-NS to fine-tune its modulatory activity (Nieto et al., 2000; Madrid et al., 2002b, 2007a; Vivero et al., 2008). In addition to modulate housekeeping genes, H-NS plays a relevant role in silencing large stretches of DNA that may have been acquired by lateral gene transfer (Lucchini et al., 2006; Navarre et al., 2006; Oshima et al., 2006). Hence, H-NS appears as a regulatory element facilitating the incorporation of horizontally acquired genes (HGT). Previous data have shed some light on the field indicating that whereas H-NS homo-oligomers modulate expression within the core genome, the preferential target of H-NS/Hha complexes are HGT genes (Baños et al., 2009). In other words, Hha-like proteins interact with H-NS allowing H-NS to discriminate between HGT and core genome and thus silencing xenogeneic DNA (Baños et al., 2009).

H-NS amino acid residues responsible for the interaction with Hha are located mainly within helices H1 and H2 of the H-NS N-terminal domain (García et al., 2006), being the arginine residue at position 12 (R12) of H-NS critical for Hha binding. Indeed, mutagenesis of amino acid R12 was shown to dramatically reduce the interaction of H-NS with Hha (García et al., 2006).

The three dimensional structure of Hha consists of four α-helical segments separated by loops (Yee et al., 2002). On the subject of the Hha protein, amino acid residues interacting with H-NS are scattered along the full length molecule (Nieto et al., 2002; García et al., 2005). Recent studies describe a non-homogeneous charge distribution of the Hha-like proteins, i.e., its positively and negatively charged residues cluster on opposing surfaces of the molecule (Paytubi et al., 2011; Ali et al., 2013). The predominantly basic surface of Hha points away from H-NS, indicating that these positively charged residues are essential for the regulatory control. This suggests that Hha could potentially provide an additional interaction surface for the nucleoprotein complex (Ali et al., 2013). On the other hand, site directed mutagenesis of conserved negatively charged residues on Hha allowed the identification of residues E25 and D48 as critical for Hha-H-NS interaction (de Alba et al., 2011). Removal of the negative charge at position 25 severely compromises the interaction with H-NS although it does not suppress it, whilst aspartic acid at position 48 is strictly required for the complex formation. The mutagenesis of aspartic acid at position 48 totally impairs the binding of Hha to the N-terminal domain of H-NS and in consequence its capability to silence *hlyABCD* expression (de Alba et al., 2011).

The lack of a clear DNA binding domain in Hha has suggested that the interactions with H-NS/StpA are required for Hha to modulate gene expression. However, it cannot be ruled out that Hha may modulate gene expression independently of H-NS/StpA. In this work we identify the set of genes of *Salmonella enterica* serovar Typhimurium SV5015 that are under the regulation of the Hha protein in an H-NS/StpA-independent manner.

## Materials and Methods

### Bacterial Strains, Plasmids, and Culture Media

Bacterial strains and plasmids used in this work are listed in **Table 1**. Cells were grown at 37°C in Luria–Bertani (LB) medium (10 g l^-1^ NaCl, 10 g l^-1^ tryptone, 5 g l^-1^ yeast extract) or LPM medium [5 mM KCl, 7.5 mM (NH_4_)_2_SO_4_, 0.5 mM K_2_SO_4_, 38 mM glycerol (0.3% v/v), 0.1% casamino acids, 8 μM MgCl_2_, 337 μM PO_4_^-3^ and 80 mM MES (for titration to pH 5.8)] (Coombes et al., 2004). The antibiotics used were kanamycin (Km) 50 μg ml^-1^, ampicillin (Ap) 100 μg ml^-1^ and chloramphenicol (Cm) 25 μg ml^-1^.

**Table 1:** Strains and plasmids used in this work.

Strains/Plasmids	Characteristics	Reference
***Escherichia coli***		
BL21 (DE3) Δ*hns*	*hsdS, gal*, (λcI*ts*857, *ind*1, *Sam*7, *nin*5, *lac*-UV5-T7 gene1) Δ*hns*::Km	Zhang et al. (1996)
DH5α	*fhuA2 lac(del)U 169 phoA glnV44 Φ80’ lacZ(del)M1*5 *gyrA96 recA*1 *relA1 endA1 thi-1 hsdR*17	Taylor et al. (1993)
***Salmonella* Typhimurium**		
SV5015	SL1344 *his*^+^	Vivero et al. (2008)
SV5015H	SV5015 Δ*hha::*Cm	Vivero et al. (2008)
SV5015S	SV5015 Δ*stpA::*Cm	Hüttener, M.
SV5015HY	SV5015 Δ*hha* Δ*ydgT::*Cm	This study
SV5015Y	SV5015 Δ*ydgT::*Cm	This study
SV5015HYS	SV5015 Δ*hha* Δ*ydgT* Δ*stpA::*Cm	This study
SV5015D	SV5015 *hha*D48N	This study
SV5015SY	SV5015 Δ*stpA* Δ*ydgT::*Cm	This study
SV5015DY	SV5015 *hha*D48N Δ*ydgt::*Cm	This study
SV5015DYS	SV5015 *hha*D48N Δ*ydgT* Δ*stpA*::Cm	This study
**Plasmids**		
pET15bHisHha	pET15b + 6xHis-*hha*; Ap^r^	Cordeiro et al. (2008)
pET15bHisHhaD48N	pET15b + 6xHis-*hha*D48N; Ap^r^	de Alba et al. (2011)
pT7-stpA	pT7-5 + *stpA*; Ap^r^	Zhang et al. (1995)
pLysS	T7 lysozyme; Cm^r^; ori p15A	Studier and Moffatt (1986)
pACYC184	Cloning vector Tc^r^, Cm^r^	Rose (1988)
pACYC184hhaSV	pACYC184 + *hha*; Cm^r^	This study
pACYC184hhaD48NSV	pACYC184 + *hha*D48N; Cm^r^	This study
pIC-ssrA2	pIC552 *ssrA*::*lacZ*, Ap^r^	Gaviria, T.
pGEM-T easy	Vector, Ap^r^	Promega
pKD4	oriR 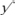 Km^r^ Ap^r^	Datsenko and Wanner (2000)
pKD3	oriR 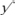 Cm^r^ Ap^r^	Datsenko and Wanner (2000)
pKD46	*oriR* 101*, rep A*101 (ts), *araBp-gam-bet-exo* (Red helper plasmid,Ts; Ap^r^)	Datsenko and Wanner (2000)
pCP20	*λc*I857 (ts), *ts-rep* (Recombinase FLP, Ts) Ap^r^, Cm^r^	Cherepanov and Wackernagel (1995)

### Genetic Manipulations and Molecular Techniques

The *hha* gene from SV5015 was amplified by PCR using the oligonucleotides pairs HhaS_BamHI and HhaS_HindIII. The generated fragment, containing the putative promoter and 67 nucleotides of the non-coding sequence downstream *hha* was cloned into pACYC184, generating plasmid pACYC184-hhaSV. The point mutation *hha*D48N was introduced into pACYC184-hhaSV using the QuikChange site-directed mutagenesis kit (Stratagene) and the pair of oligonucleotides SalD48N_For/SalD48N_Rev, resulting in plasmid pACYC184-hhaD48NSV. The chromosomal *hha*D48N point mutant in strain SV5015 was constructed using the “stitch PCR” technique. This technique was used to “stitch” two DNA fragments together, the *hha*D48N and the kanamycin resistance genes. The *hha*D48N was PCR amplified from plasmid pACYC184-hhaD48NSV using primer pairs #1/#2. The kanamycin resistance cassette flanked by FRT sites was PCR amplified from plasmid pKD4 separately using oligonucleotides #3 and #4. The two PCR products were annealed at their overlapping regions and amplified as a single fragment using oligonucleotides pairs #1/#4 (for details see Supplementary Figure S1). The annealed product was purified, cloned into pGEM-T easy vector (Promega) after the addition of an A-tail and electroporated into *E. coli* DH5α. Sequencing analysis confirmed the correct amplification and the “stitch” fragment was amplified from the plasmid using oligonucleotides #1 and #4. Finally, the chromosomal *hha*D48N point mutant (SV5015D) was obtained using the procedure described by (Datsenko and Wanner, 2000). Briefly, the PCR product was *Dpn*I-digested, purified and used to electroporate strain SV5015 carrying the plasmid pKD46 grown at 30°C in the presence of 10 mM arabinose. Recombinant clones were selected at 37°C in LB medium containing kanamycin, tested for the absence of pKD46, and confirmed by PCR. The chromosomal deletion of *ydgT* gene was generated by the λ Red recombinant method as previously described (Datsenko and Wanner, 2000) resulting in strain SV5015Y. Oligonucleotides YdgT_P1 and YdgT_P2 were used to amplify chloramphenicol resistance gene from pKD3 plasmid. All the recombinants obtained were checked by PCR with YDGT-P1UP and YDGT-P2DOWN oligonucleotides.

When needed, the kanamycin and chloramphenicol resistance genes were removed using pCP20 plasmid (Cherepanov and Wackernagel, 1995). SV5015Y strain was used as a donor to transduce the *ydgT::Cm* mutation into strains SV5015H, SV5015D, and SV5015S using phage P22 HT (Sternberg and Maurer, 1991) generating strains SV5015HY, SV5015DY, and SV5015SY, respectively. Additionally, SV5015S strain was used to transfer the *stpA::Cm* mutation to the double mutant strains, obtaining strains SV5015HYS and SV5015DYS. The sequence of all oligonucleotides used in this work is indicated in **Table 2**.

**Table 2:** Oligonucleotides used in this work.

Oligonucleotides	Sequence 5′–3′
HhaS_BamHI	GACGGATCCCAAAAATGGCGTAAATCGG
HhaS_Hindlll	CGGAAGCTTGCCCGTTGTGTTATTAGCC
SalD48N_For	GTATTTTACTCAGCTGCGAATCACCGTCTTGCAGAATTG
SalD48N_Rev	CAATTCTGCAAGACGGTGATTCGCAGCTGAGTAAAATAC
#1	TTACAATCATAGGTAGAATTTATGTCTGATAAACCATTAA
	CTAAAACTGATTATTTGATGC
#2	GAAGCAGCTCCAGCCTACACGAACGAGGAGGCAGATAAC
	ACCTGCGTGTTCTCTAAAAAG
#3	GTGTTATCTGCCTCCTCGTTCGTGTAGGCTGGAGCTGCTT
	CGAAGTTCCTATACTTTCTA
#4	CTATATCACTGTTCTATAATAGCCCGTTGTGTTATTAGCC
	ACATATGAATATCCTCCTTAG
YdgT_P1	GTTTATTTTTTATCAGTGACTACTCCGTTGGCATTATATTT
	AATGTGTAGGCTGGAGCTGCTTC
YdgT_P2	GGGGCAAATATTATAAGGTTTTTGATGTTAAACGCTACTT
	TCTCATATGAATATCCTCCTTAGT
YdgT_P1UP	CCTGACTCTTTACCGGTAAG
YdgT_P2DOWN	GTAGTCATATCTTCTCCGGG
ssrA_qPCR_F	GCTCAATCTCAAGAATACGC
ssrA_qPCR_R	CTGCCGTTTCTGAACCATTG
sipB_qPCR_F	TTAGATAAGGCCACGGATGC
sipB_qPCR_R	CCTGGGAAACCTGATTCTGA
motB_qPCR_F	GATTTCCATCTCCAGCCCTA
motB_qPCR_R	GCTGTTGGGTGTAATCATCG

### Pull-Down Experiments

BL21 (DE3) *Δhns* cells carrying plasmids pET15bHisHha, pET15bHisHhaD48N, or pLysS/pT7-stpA, were grown in 500 ml of LB at 37°C with shaking until OD_600nm_ of 0.4. Following induction with 0.5 mM isopropyl-β-D-thiogalactopyranoside (IPTG), cells were grown for 2 h under the same conditions. Cells were then harvested by centrifugation at 11000 × *g* at 4°C for 30 min and resuspended in 20 ml of lysis buffer A (20 mM HEPES pH 7.9, 100 mM KCl, 5 mM MgCl_2_, 50 mM imidazole and 10 glycerol). Lysis was carried out by sonication. To obtain a clear lysate the extracts were centrifuged at 35000 × *g* at 4°C for 20 min. His-tagged Hha proteins were purified with 0.5 ml Ni^2+^-NTA beads (Qiagen) as previously described (Nieto et al., 2000).

### Gel Electrophoresis and Western Blot

Protein samples were analyzed by SDS-PAGE and stained with Coomassie brilliant blue or immunoblotted by western blot upon transfer of proteins to PDVF membranes. Western blot analysis was performed with polyclonal antibodies raised against *E. coli* StpA protein [1:2000] (Johansson and Uhlin, 1999). Horseradish peroxidase-conjugated goat anti-rabbit IgG [1:100000] (Sigma) was used as secondary antibody. Immunodetection of the transferred proteins was performed by enhanced chemiluminescence using the software Quantity One (Bio-Rad).

### Microarray Analysis

Total RNA was isolated from three independent cultures of the strains SV5015, SV5015H, SV5015D and SV5015S grown at 37°C in LB to an OD_600_ of 0.6. The RNA was purified as previously described (Paytubi et al., 2014). Transcriptomic analyses were performed on a custom DNA microarray engineered by Nimblegen. The custom Nimblegen microarray contained 4941 probes (4519 SL1344, 103 SL1344_pSLT, 99 SL1344_pRSF, 14 SL1344_pCOL1B, 206 R27) from the genome sequence of *S. enterica* serovar Typhimurium SL1344 (Paytubi et al., 2014). Retrotranscription, labeling, hybridization, microarray scanning, and data analysis were performed as recommended by Nimblegen standard protocol. These transcriptomic experiments and the statistical analysis of the microarray data were carried out at Institute of Biomedical Research, Barcelona.

The complete data set has been deposited under accession number E-MTAB-3621 at http://www.ebi.ac.uk/arrayexpress.

### qRT-PCR

qRT-PCR analysis was performed to corroborate the microarray data (Supplementary Table S3) using strains SV5015H, SV5015D and SV5015S, SV5015HY, and SV5015DY versus wild-type (WT) strain, SV5015. Real-time quantitative reverse transcription-PCR was used to confirm microarray results and analyze expression of the *ssrA*, *sipB*, and *motB*. Briefly, 1 μg of total RNA was reverse transcribed to generate cDNA using the “High-capacity cDNA Reverse Transcription kit” (Applied Biosystems) as recommended by the manufacturer. As a control, parallel samples were run in which reverse transcriptase was omitted from the reaction mixture. Real-time PCR using “Power SYBR Green PCR Master Mix kit” (Applied Biosystems) was carried out on the StepOne Real-Time PCR System Thermal Cycling Block (Applied Biosystems). Oligonucleotides complementary to the genes of interest were designed using Primer3 online tool provided by the Whitehead institute^1^. Expression levels of the tested genes were normalized to the reference strain (SV5015) as in Ali et al., 2013.

### Siderophore Detection

A colorimetric assay, Siderotec Assay^TM^ (Emergenbio), was used for the detection of the siderophores secreted by strain SV5015 and its derivatives. Cultures of each strain were grown in LB at 37°C until the beginning of stationary phase (OD_600nm_ 2.0). One-milliliter of each culture was centrifuged and the supernatant was used for siderophores detection. The assay was performed as indicated by the supplier with the following modification. To enhance the detection, the standard solution was added to the recommended mix in the following proportion: 10 μl catalyst, 90 μl dye reagent, and 20 μl of the standard. Eighty-microliter of each strain supernatant was added to the mixture and the colorimetric changes were determined by measuring the OD_630nm_ of the samples, as recommended by the supplier.

### Swimming Motility

Swimming motility was performed on Tryptone broth (TB) plates (1% tryptone and 0.5% NaCl) containing 0.35% agar. Overnight bacterial cultures grown in LB at 37°C were spotted (5 μl) on the center of the plates. The colony diameter was measured after incubation for 8 h at 37°C.

### Transmission Electron Microscopy

Bacterial strains used for flagella visualization were obtained from motility plates grown overnight at 37°C. A slice of the motility perimeter of the indicated strains was collected, resuspended in filtered Ringer ¼ solution and centrifuged at 1500 × *g* for 5 min. Cu-Carbon grid (CF200-Cu Carbon Film On 200 Mesh Copper Grids, Electron Microscopy Sciences) was soaked for 60 s on a 5 μl drop of each strain, washed three times with water for 20 s and stained for 60 s using a 2% (w/v) uranyl acetate solution (Polysciences). Once stained, the grids were dried for at least 24 h before visualization under a JEOL JEM1010 transmission electron microscope. Images were obtained using the software analysis (Soft Imaging System GmbH, Münster, Germany). Each sample was observed for at least 100 cells.

### β-Galactosidase Activity

The reporter gene fusion *ssrA*::*lacZ* from plasmid pIC-ssrA2 was used to evaluate *ssrA* transcriptional expression. β-galactosidase activity assays were performed as previously described (Miller, 1993). Strains were grown at 37°C with shaking in LB until reaching an OD_600nm_ of 0.6 (as in the transcriptomic experiments) or in LPM pH 5.8 culture medium, which induces SPI-2 gene expression as previously described (Coombes et al., 2004; Silphaduang et al., 2007).

### Macrophage Survival Assay

RAW 264.7 murine macrophages (ATCCTIB-71), were grown in Dulbecco’s modified Eagle medium (DMEM) containing 10% heat-inactivated fetal bovine serum (HI FBS), x1 MEM non-essential amino acids and 2 mM L-glutamine in a humidified atmosphere (37°C in 5% CO_2_). *S.* Typhimurium strains were grown in LB to stationary phase and opsonized for 30 min in DMEM containing 10% normal mouse serum (Charles River Laboratories; Balb/c mouse male). Bacteria were then centrifuged onto macrophages seeded in 6-well tissue culture plates at a multiplicity of infection (MOI) of 10:1 and incubated for 30 min. After infection, macrophages were washed twice with DPBS (Dulbecco’s Phosphate-Buffered Saline) and incubated for 90 min more in medium containing 100 μg ml^-1^ gentamicin to kill the remaining extracellular bacteria (2 h post-infection, corresponding to time 0). For the remainder of the experiment, medium containing 10 μg ml^-1^ gentamicin was used to prevent extracellular bacterial replication. At 2 and 16 h post-infection time points, infected macrophages were washed twice with DPBS and lysed with 0.1% Triton X-100 in DPBS. Appropriate serial dilutions of the lysates were plated onto LB agar to enumerate colony-forming units. Intracellular replication ratio (16 h versus 2 h) was calculated.

## Results

### HhaD48N Interacts with StpA

**FIGURE 1:**
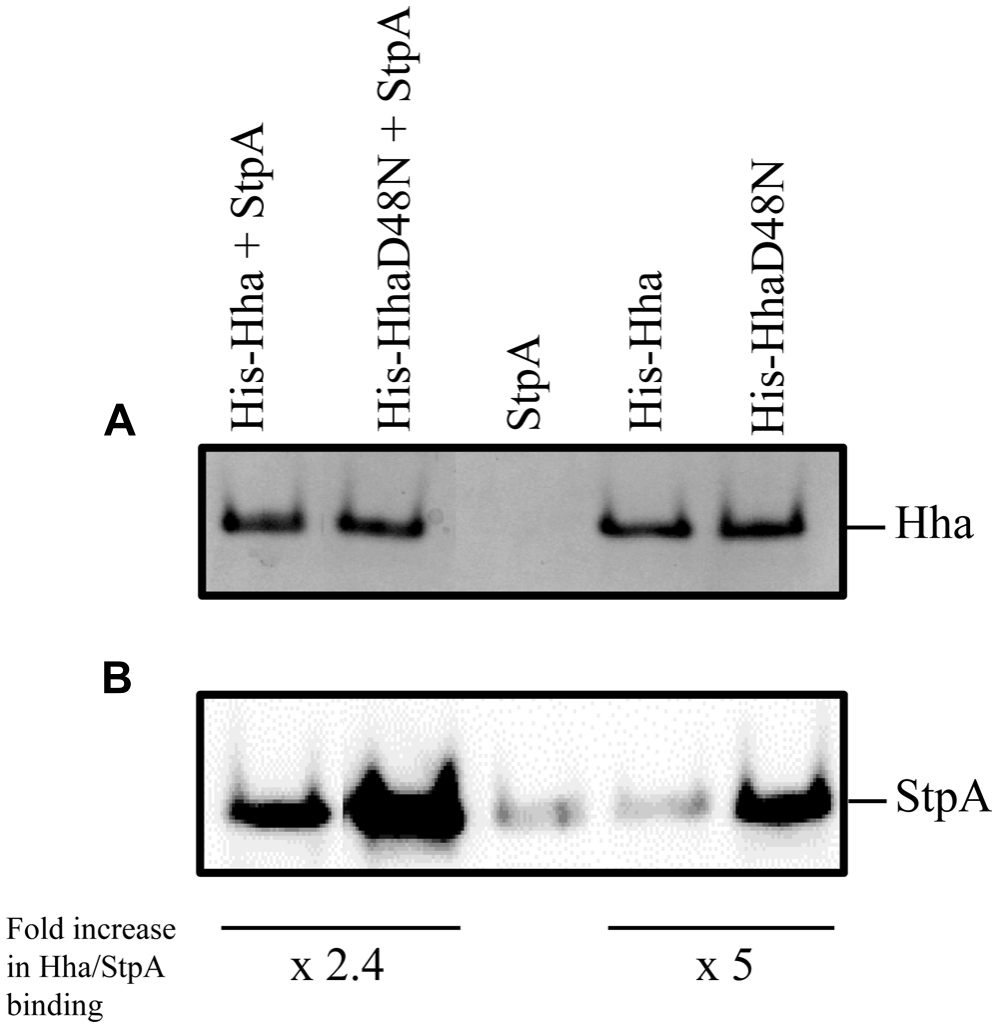
**Pull-down experiments.** His-Hha and His-HhaD48N were purified from whole cell extracts using Ni^2+^-NTA beads. **(A)** Coomassie blue stained SDS-PAGE loaded with the first eluted fraction from the Ni^2+^-NTA agarose matrix after binding of the indicated total cellular extracts. **(B)** Immunodetection of StpA protein from the same samples as in **(A)**. The relative amount of StpA was normalized to that of the corresponding Hha variant in each sample pair and fold increase in Hha/StpA binding is indicated.

In order to investigate the specific regulatory role of Hha protein independently of its interaction with H-NS, we decided to introduce the D48N mutation in the Hha protein of *S.* Typhimurium SV5015 strain, generating then a mutant derivative not capable to form Hha/H-NS complexes while maintaining the Hha structure (de Alba et al., 2011). Presumably, Hha residues involved in StpA interaction are the same as for the H-NS protein. However, to rule out that the effects of the *hha*D48N mutation might be caused by a possible interaction with StpA, pull-down experiments using His-tagged Hha and HhaD48N proteins were performed. To avoid the interaction of the WT Hha protein and H-NS, which could mask the interaction with StpA, an Δ*hns* genetic background was used. His-Hha, His-HhaD48N and StpA proteins were overexpressed using plasmids pET15bHisHha, pET15bHisHhaD48N, and pT7-stpA, respectively, in *E. coli* BL21 (DE3) Δ*hns* (His-Hha and His-HhaD48N) or *E. coli* BL21 (DE3) Δ*hns* pLysS (StpA). Ten-milliliter of clarified supernatants containing His-Hha or His-HhaD48N recombinant proteins were mixed with 10 ml of clarified supernatants containing overexpressed StpA and altogether was coated onto a Ni^2+^-NTA matrix as described in the “Materials and Methods” section. His-tagged Hha variants were eluted with 200 mM imidazole and tested for the presence of the Hha variants and the StpA protein by SDS-PAGE followed by Coomassie staining or western blot, respectively (**Figure 1**). Unexpectedly, the results obtained showed that HhaD48N (“H-NS blind” Hha mutant) still supported the interaction with the StpA protein. The amount of immunodetected StpA protein that coeluted with HhaD48N was more than twofold higher than that detected for the WT Hha. Similar results were obtained when His-tagged proteins were purified directly from cellular extracts containing either overexpressed His-Hha, His-HhaD48N, or StpA. As shown in the StpA control lane, a low level of StpA was detected due to non-specific binding of the protein to the beads. However, when HhaD48N was overexpressed and purified, endogenous StpA clearly coeluted up to fivefold compared to the WT Hha.

### Hha Protein Regulates a Set of Genes Independently of H-NS/StpA

The above reported data show that cells expressing the mutant HhaD48N protein can be used to assess whether Hha is able to modulate gene expression independently of its interaction with H-NS in *S.* Typhimurium. To investigate this, transcriptomic analyses of the WT strain *S.* Typhimurium SV5015, the Δ*hha* (SV5015H) and the point mutant *hha*D48N strain (SV5015D) were carried out. Having in mind that HhaD48N protein interacts with StpA, the Δ*stpA* strain (SV5015S) was included in the transcriptomic study to discard possible effects on gene regulation caused by this interaction. The transcriptome of each mutant was compared to that of the WT strain (**Table 3** and Supplementary Table S1). The *hha* mutation caused a more than twofold differential expression of 659 genes (406 up-regulated and 253 down-regulated). In the case of the *hha*D48N mutant, 499 genes were deregulated (310 up-regulated and 189 down-regulated). Finally, the largest effect was found in the Δ*stpA* mutant, which showed an altered expression of 783 genes (423 up-regulated and 360 down-regulated). For all mutant strains, more genes were up-regulated than down-regulated, indicating that these proteins play an important role in gene expression acting mainly as transcriptional repressors.

**Table 3:** Total and relative number of genes deregulated (more than twofold, *p*-value <0.05) in strains SV5015H, SV5015D and SV5015S, versus wild-type strain, SV5015.

	SV5015H vs. SV5015		SV5015D vs. SV5015		SV5015S vs. SV5015	
	Up-regulated	Down-regulated	Up-regulated	Down-regulated	Up-regulated	Down-regulated
Chromosome (4527)	386 (8.5%)	251 (5.5%)	293 (6.4%)	189 (4.1%)	409 (9%)	356 (7.8%)
pSLT (103)	11 (10.6%)	2 (1.9%)	9 (8.7%)	n.d	9 (8.7%)	n.d
pCollB (100)	9 (9%)	n.d	8 (8%)	n.d	5 (5%)	4 (4%)

*n.d., not detected*.

To further unravel the Hha regulon in *Salmonella*, we considered the following. Genes whose expression is altered in an Δ*hha* strain would correspond to both (i) genes whose expression is dependent of Hha through its interaction with H-NS, and (ii) the subset of genes regulated by Hha autonomously of this interaction. On the other hand, the genes showing an altered expression in an *hha*D48N strain are those regulated by the Hha/H-NS complex. Thus, the genes showing a deregulated expression in an *hha* mutant (Hha and Hha/H-NS dependent), but unaffected in an *hha*D48N mutant (Hha/H-NS dependent) or in an *stpA* mutant (Hha/StpA dependent), are good candidates to be regulated by Hha independently of its interaction with H-NS and StpA.

**FIGURE 2:**
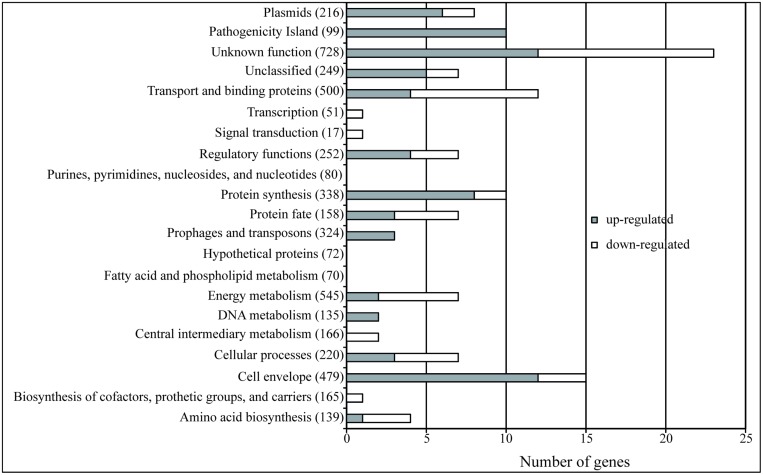
**The Hha regulon**. The bars indicate the number of genes of each category that show an altered expression in an Δ*hha* mutant independently of its interaction with H-NS or StpA. The gray bars indicate the number of genes that are up-regulated (FC > 2, *p-*value <0.05) and the white bars indicate the down-regulated genes (FC < –2, *p-*value <0.05). The total number of genes belonging to each of the functional categories (JCV Institute) is specified in parenthesis.

Consequently, when looking more deeply into the transcriptomic data, we were able to identify 120 genes as regulated by Hha independently of H-NS/StpA (Supplementary Table S2). Seventy-three of these genes were up-regulated and 47 down-regulated. In order to determine the global pattern of gene regulation held exclusively by Hha, the differentially expressed genes were grouped in their functional categories (J. Craig Venter Institute; **Figure 2**). The functional categories that showed the highest number of Hha-dependent genes correspond to genes of unknown function, pathogenicity islands, cell envelope, transport, and binding of proteins and protein synthesis. It is noteworthy that the number of pathogenicity island genes affected represents more than 10% of the total number of genes that belong to this category, resulting in the category with the highest percentage of affected genes.

### Regulation of Genes Associated to Iron Transport by the Hha Protein

*fepG*, *fepD*, *fepB*, and *entS* genes are presumably up-regulated, directly or indirectly, by Hha in an H-NS-independent fashion (**Table 4**). The products of all these genes are involved in iron transport. FepG and FepB form, together with FepC, an ABC transporter of siderophores (Zhu et al., 2005; Crouch et al., 2008). EntS is a transmembrane protein related to enterobactin secretion (Furrer et al., 2002; Methner et al., 2008).

**Table 4:** Genes related to iron transport, motility, and chemotaxis and pathogenicity islands that are regulated by the Hha protein in an H-NS/StpA-independent manner.

Gene	ORF	Function	FC(SV5015H vs SV5015)
**Iron transport**	
*fepG*	SL0579	Ferric enterobactin transport system permease protein	-2.1
*fepD*	SL0580	Ferric enterobactin transport system permease protein	-2.6
*fepB*	SL0582	Ferrienterobactin-binding periplasmic protein	-2.3
*entS*	SL0581	Enterobactin exporter	-2.3
**Motility and chemotaxis**		
*flgN*	SL1108	Flagella synthesis protein	-2.3
*motB*	SL1857	Motility protein B	-2.1
*tcp*	SL3542	Methyl-accepting chemotaxis citrate transducer	-2.3
*tsr*	SL4464	Methyl-accepting chemotaxis protein I	-2.3
**Pathogenicity islands**		
*pipA*	SL1026	Pathogenicity island encoded protein (SPI-5)	2.8
*sigE*	SL1029	Pathogenicity island-encoded protein; cell invasion protein (SPI-5)	3.3
*sigD*	SL1030	Pathogenicity island-encoded protein; Type III secretion system effector protein (SPI-5)	3.2
*ssrB*	SL1325	Two-component response regulator (SPI-2)	2.7
*ssrA*	SL1326	Two-component sensor kinase (SPI-2)	2.7
*sseG*	SL1339	Type III secretion system effector protein-modulates the positioning of the SCV (SPI-2)	4.0
*ssaQ*	SL1352	Type III secretion system protein (SPI-2)	2.7
*sipC*	SL2863	Translocation machinery protein (SPI-1)	2.4
*sipB*	SL2864	Translocation machinery protein (SPI-1)	2.5
*siiE*	SL4197	Large repetitive protein (SPI-4)	3.5

**FIGURE 3:**
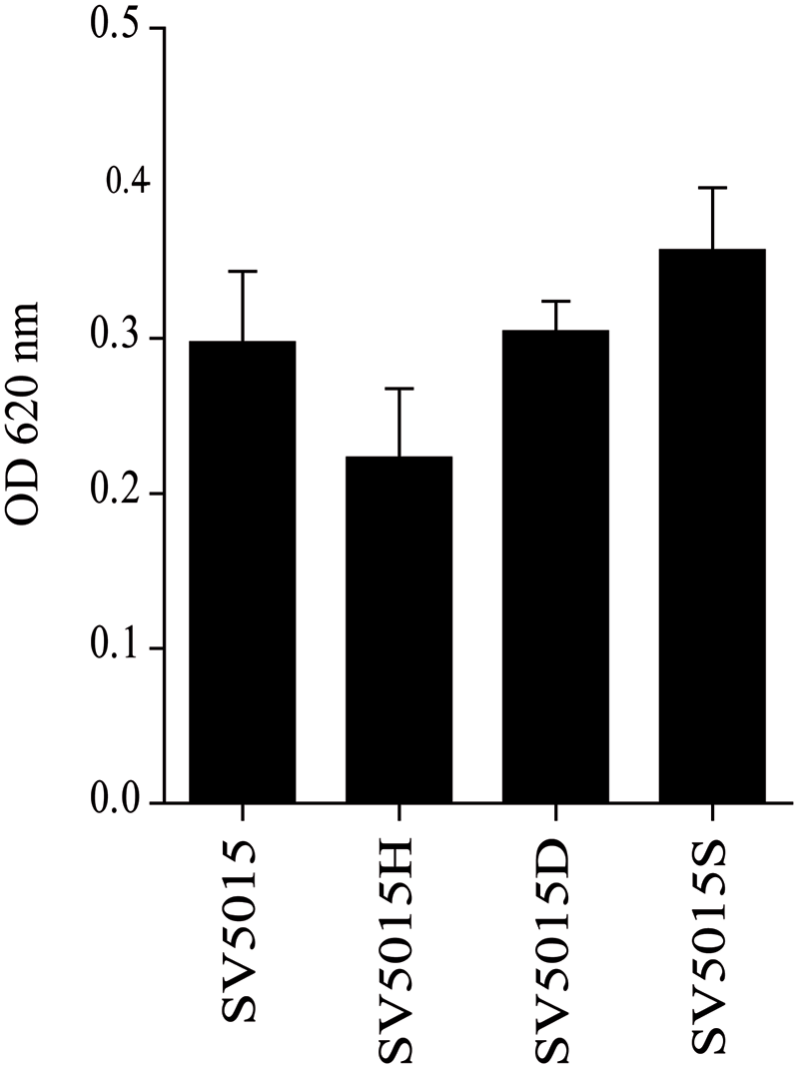
**Detection of siderophores in culture supernatants of strains SV5015, SV5015H, SV5015S and SV5015D.** Error bars represent the SD of three independent experiments.

In light of the above, we tested the role of the Hha protein in the synthesis and transport of siderophores. To this end, the presence of siderophores in bacterial culture supernatants of strains SV5015, SV5015H, SV5015D, and SV5015S was determined (**Figure 3**). As expected, the level of siderophores detected in the supernatant of SV5015H strain was 25% lower compared to the WT strain. Despite these results are not statistically significant, the data are consistent with the changes observed in the transcriptomic data. In contrast, the level of siderophores detected in supernatants of strains SV5015D and SV5015S was similar or even higher than in the WT strain, indicating that the interaction of Hha with H-NS/StpA is not required to regulate the genes associated to iron transport.

### Hha Controls Motility at the Motor Level

The transcriptomic data allowed us to identify four genes involved in motility and chemotaxis, *flgN*, *motB*, *tcp*, and *tsr*, that are down-regulated in an *hha* mutant (**Table 4**). FlgN is a chaperone related to the secretion of hook-associated proteins FlgK and FlgL. MotB forms, together with MotA, a transmembrane proton-channel that drives flagellar rotation (Morimoto and Minamino, 2014). Tcp and Tsr are chemoreceptors located in the cytoplasmic membrane (Yamamoto and Imae, 1993; Okumura et al., 1998; Iwama et al., 2006).

**FIGURE 4:**
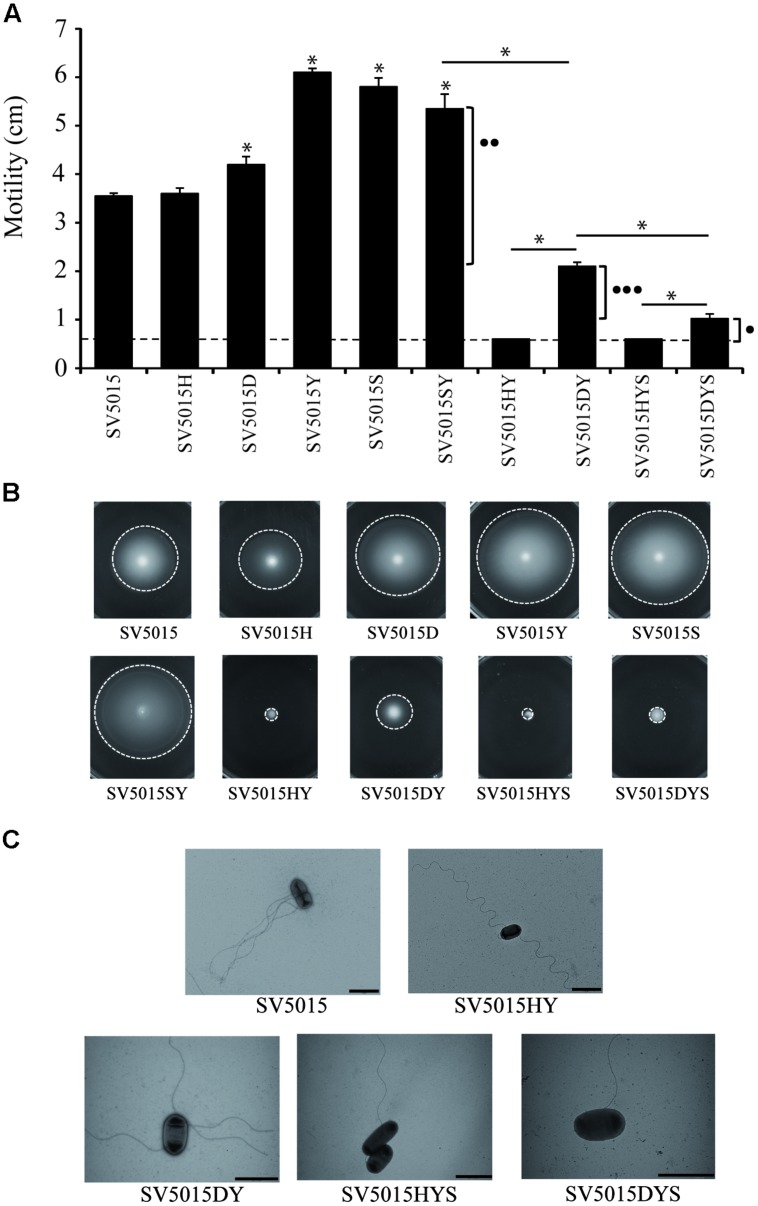
**Motility assays.** Swimming of different strains assayed after 8 h incubation at 37°C. **(A)** The diameter of the bacterial growth was measured. Dashed lines indicate the diameter of growth of the inoculated strain. The effects on motility of the Hha protein interacting with H-NS (••) or StpA (•••) or independently of interactions with H-NS or StpA (•) are indicated. The error bars represent the SD of three independent experiments. The significance of the comparison between the wild-type (WT) strain and single mutant strains is shown with an asterisk (*p-*value <0.05), and the significance between mutant strains is indicated with a bar and an asterisk (*p-*value <0.05). **(B)** Representative motility agar plates are shown. Dashed lines indicate the limit of bacterial motility. **(C)** Bacteria and surface flagella were stained using 2% uracil acetate solution and visualized using transmission electron microscopy. The images are representatives of the phenotypes observed.

The effect on genes contributing to bacterial chemotaxis and flagellar function was confirmed by a motility assay (**Figure 4**). YdgT, the paralog of Hha, appears to fulfill some of the functions of the Hha protein (Paytubi et al., 2004). Moreover, it has been previously described that the *hha ydgT* double mutant completely abolishes the motility of *S.* Typhimurium SV5015 (Vivero et al., 2008). Consequently, we decided to include *ydgT* mutant strains in the motility assays. By using strains deficient in YdgT, we intended to withdraw any possible effect of the YdgT-H-NS/StpA interaction that could potentially mask the effect of Hha on motility.

Cultures of SV5015 and its mutant derivatives were spotted on TB agar plates and the colony diameter was measured. The results obtained (**Figures 4A,B**) showed that the *hha* mutation does not cause a significant effect on motility, compared to the WT strain. In contrast, single SV5015D, SV5015Y, SV5015S, and double SV5015SY mutations cause a slight increase in motility compared to the SV5015 strain (1.2-, 1.7-, 1.6-, and 1.5-fold, respectively). As expected, the SV5015HY strain is totally impaired in motility as previously described (Vivero et al., 2008; Wallar et al., 2011), as well as the SV5015HYS triple mutant strain. Remarkably, the double mutant SV5015DY only shows a 1.7-fold decrease in motility compared to the WT strain. qRT-PCR analysis of strains SV5015H, SV5015D, SV5015S, SV5015HY, and SV5015DY versus WT strain SV5015 using primers against *motB* gene confirmed these results (Supplementary Table S3). Despite that this strain does not completely recover the motility showed by strain SV5015, the results suggest that Hha plays a role in motility in an H-NS-independent manner. The phenotype shown by this double mutant strain could still be due to the effect of the interaction between HhaD48N and StpA. To bypass this effect, a triple mutant *hha*D48N *ydgT stpA* (SV5015DYS) was used. A twofold decrease in motility was observed, compared to the SV5015DY strain, although the motility is not completely abolished as it occurs in the *hha ydgT stpA* (SV5015HYS) strain. This result indicates that the interaction between Hha and H-NS/StpA is important for the regulation of expression of genes related to motility (as indicated in **Figure 4A**). Nevertheless, the fact that the presence of an “H-NS blind” Hha mutant causes an increased motility phenotype in the absence of YdgT (SV5015DYS compared to SV5015HYS), indicates that Hha also plays a role in motility in an H-NS/StpA-independent manner.

*Trans*-complementation assays were performed to confirm the role on motility of the Hha protein (Supplementary Figure S2). The triple mutation SV5015HYS totally abrogates the motility of SV5015 strain which is completely recovered when complemented in *trans* with the WT *hha* gene cloned in plasmid pACYC184. Contrarily, when complementing the non-motile phenotype with the “H-NS blind” Hha mutant, a partial recovery of the motility is obtained, corresponding to the regulatory role in motility that Hha protein carries out independently of its interaction with H-NS or StpA.

To discern whether the absence of motility of strains SV5015HY and SV15015HYS was due to a defect on flagellar production or flagellar rotation, cells of the different strains grown on motility plates were observed using transmission electron microscopy. Strains SV5015HY and SV5015HYS (non-motile strains) presented flagella on their surface, although to a less extend than strains SV5015, SV5015DY, and SV5015DYS, which showed motile phenotype in different degrees (**Figure 4C**). Results suggest that the observed absence or decrease in motility might rely on genes involved in motility at the motor level, more than to a decrease of flagella.

### Role of Hha Protein on the Regulation of Expression of SPI Genes

**FIGURE 5:**
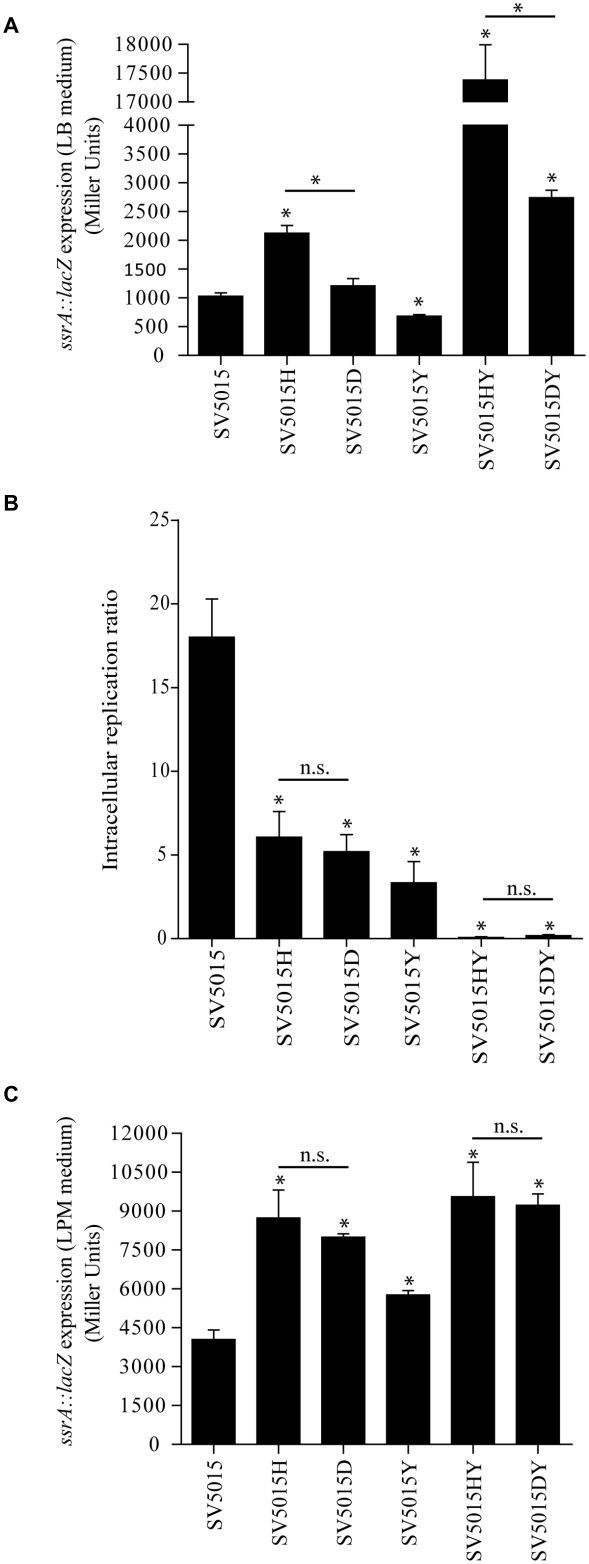
**Role of the Hha protein in SPI-2 regulation.** β-galactosidase activity of a *ssrA::lacZ* fusion in LB medium **(A)** and LPM medium **(C)** of SV5015, SV5015H, SV5015D, SV5015Y, SV5015HY and SV5015DY. **(B)** Intracellular replication ratio after infection of RAW 264.7 murine macrophages (16 h vs. 2 h) with the above mentioned strains. The error bars represent the SD of three independent experiments. The significance of the comparison between the WT strain and SV5015 mutant strains is shown with an asterisk (*p-*value <0.05); the significance between mutant strains is indicated with a bar and an asterisk (*p-*value <0.05) and n.s. stands for not statistically significant.

The transcriptomic data reveals that the Hha protein is engaged in the regulation of some genes related to pathogenicity islands independently of its interaction with H-NS or StpA (**Table 4**). Among these, *ssrA* and *ssrB* genes were found to be up-regulated. These genes encode the two-component regulatory system SsrA–SsrB responsible for the activation of expression of the SPI-2 genes (Tomljenovic-Berube et al., 2010; Xu and Hensel, 2010; Osborne and Coombes, 2011). It has been described that the expression of SPI-2 genes is essential for the intracellular survival and replication of *Salmonella* within macrophages (Schmidt and Hensel, 2004; Fàbrega and Vila, 2013). Moreover, Hha and YdgT proteins play a regulatory role in the expression of virulence factors encoded in SPI-2 (Coombes et al., 2004; Silphaduang et al., 2007; Vivero et al., 2008), although it is not clear whether this modulatory role depends upon their interaction with H-NS. The transcriptomic data revealed that the above mentioned role of Hha protein could be H-NS-independent, since *ssrA* and *ssrB* genes showed an altered expression in an *hha* mutant whereas the *hha*D48N mutation did not cause a significant deregulation of the gene expression when compared to the WT strain. A transcriptional fusion of the *ssrA* promoter with the *lacZ* reporter gene into the multicopy plasmid pIC552, pIC-*ssrA2,* was used to determine the transcriptional level of expression of the *ssrA* gene in different genetic backgrounds (**Figure 5A**). The strains were grown in LB medium at 37°C to an OD_600_ of 0.6, the same conditions as in the transcriptomic assay. In agreement with the transcriptomic data, the *hha* mutation causes an increase of the expression of the *lacZ* gene depending on the *ssrA* promoter (twofold). In contrast, expression from the *ssrA* promoter in strains SV5015D or SV5015Y was similar to the values obtained for the WT strain. Since the YdgT protein can compensate the effect of an *hha* mutation, we expected a more notorious regulatory role of Hha in a *ydgT* genetic background. Interestingly, the expression from the *ssrA* promoter in the double mutant strain SV5015HY, showed a drastic increase (17-fold) of the expression. In striking contrast, in the double mutant strain SV5015DY, the *lacZ* expression only showed a 2.6-fold increase compared to the WT strain. qRT-PCR analysis of strains SV5015H, SV5015D, SV5015S, SV5015HY, and SV5015DY compared to strain SV5015 to assess transcript levels of *ssrA* confirmed these results (Supplementary Table S3). Results showed that Hha protein might play a role by its own in the regulation of SsrA–SsrB two component system, and thus in the expression of SPI-2 genes.

The fine tuning of the SPI-2 gene expression contributes to the survival and replication of *Salmonella* within macrophages. Considering the results obtained, we would expect different behavior when testing replication of the different genetic background strains within macrophages. To verify the significance of the Hha protein on the regulation of the two component system SsrA–SsrB and thus on the pathogenicity of *Salmonella*, we assessed the ability of the same strains used in the β-galactosidase assay to replicate in macrophages (**Figure 5B**). All single mutant strains (SV5015H, SV5015Y and SV5015D) showed a reduced intracellular replication in the murine macrophage cell line RAW 264.7 when compared to the WT strain SV5015. This result is consistent with previous reported results concerning the role of Hha and YdgT in the SPI-2 regulation (Coombes et al., 2005; Silphaduang et al., 2007). However, these results are not consistent with the expression levels of *ssrA* observed both in the transcriptomic data and the β-galactosidase assay under the conditions used (LB medium). The data obtained in the macrophages replication assay indicates that the Hha protein does not play a role independent of H-NS in the replication of *Salmonella* within macrophages. It has been documented that the LPM medium (low phosphate and low magnesium, pH 5.8) mimics the physiological conditions that induce the expression of SPI-2 genes (Coombes et al., 2004; Silphaduang et al., 2007). To confirm whether the culture medium has an effect on the regulation of *ssrA*, held by Hha itself or with H-NS, we decided to analyze the transcriptional expression of *ssrA* in cells growing in SPI-2-inducing medium. **Figure 5C** shows the results obtained. When using LPM medium, the levels of β-galactosidase activity shown by strains *hha* and *hha*D48N are not significantly different, although in both cases are approximately twofold higher than the activity detected for the WT strain. In contrast to the data obtained when cells were grown in LB medium, the double mutant strains (SV5015HY and SV5015DY) do not show differences in the expression levels of *lacZ* compared to the respective single mutant strains (SV5015H and SV5015D). Strikingly, the β-galactosidase activity determined in the LPM medium was significantly similar in both double mutant strains. These results are consistent with the data obtained in the replication assay within macrophages.

In short, we suggest that Hha could play a “dual” regulatory role in the *ssrA* expression, based on its dependence of H-NS. The Hha-mediated repression of *ssrA* expression coulfd be H-NS-independent in extracellular conditions while H-NS-dependent during intercellular growth.

## Discussion

Hha, a nucleoid- associated protein, was first identified as a modulator of the expression of hemolysin in *E. coli* (Nieto et al., 1991; Carmona et al., 1993; Fahlen et al., 2000; Sharma and Zuerner, 2004). Furthermore, Hha-like proteins have been related to the environmental regulation of virulence factors in several enterobacteria (Mouriño et al., 1996; Madrid et al., 2002a,b). It has been described that the Hha protein affects gene expression through its interaction with H-NS (Nieto et al., 2000; Forns et al., 2005; Ali et al., 2013; Aznar et al., 2013) and this interaction is common to other members of both families (Hha/StpA, YdgT/H-NS, YdgT/StpA, YmoA/H-NS; Nieto et al., 2002; Paytubi et al., 2004). As a result of the interaction with H-NS, Hha can alter the target specificity of H-NS playing a significant role in the recognition of HGT sequences (Baños et al., 2009; Ali et al., 2013; Aznar et al., 2013).

Several studies have suggested the modulatory role of Hha in enterobacteria through its interaction with DNA regulatory sequences (Fahlen et al., 2001; Sharma and Zuerner, 2004; Sharma et al., 2005; Sharma and Bearson, 2013; Sharma and Casey, 2014). It is worth to remark that only in one of these reports (Olekhnovich and Kadner, 2007) the Hha protein has been purified in an Δ*hns* genetic background. Bearing in mind that Hha interacts with H-NS, and that H-NS copurifies with Hha, it is likely that the DNA binding activity reported in most of these studies is due to Hha/H-NS complexes.

The H-NS protein has two different domains, the N-terminal domain involved in oligomerization, and the C-terminal domain responsible for its binding to the DNA. The Hha protein shows a high degree of homology with the N-terminal domain of H-NS and it has been proposed that Hha-like proteins might have evolved to mimic the N-terminal domain of H-NS (Madrid et al., 2007a). Despite the fact that the Hha protein does not have defined domains and most of the protein sequence contributes to its interactions with H-NS (Nieto et al., 2002), it shows a dipolar distribution. Positively and negatively charged residues cluster on opposing surfaces of the molecule showing an asymmetrical charge distribution that reveals specific functions (Paytubi et al., 2011; Ali et al., 2013). Conserved positively charged residues on the surface of Hha are positioned in the same orientation as predicted for the DNA binding domain of H-NS, whilst a combination of basic and acidic Hha residues interacts with H-NS. NMR analysis focused on the study of the function of the acidic residues of the Hha protein in the interaction with H-NS, allowed the identification of aspartic acid in position 48 as essential for this interaction (de Alba et al., 2011). Consistent with this finding, the structure resolved by Ali et al. (2013) confirmed that these residues are located within the Hha/H-NS (1–46) interaction interface.

StpA is the best characterized paralog of H-NS (Zhang and Belfort, 1992; Zhang et al., 1996; Johansson and Uhlin, 1999; Lucchini et al., 2009). Structurally, H-NS and StpA are similar and both have a tightly relationship with the Hha family of proteins. Although the specific residues of StpA involved in the interaction with Hha have not been identified, attending to its similarity with H-NS, it is reasonable to speculate that such interactions might comprise the same residues described for the H-NS/Hha interaction (García et al., 2006; de Alba et al., 2011). Pull-down experiments of StpA with Hha or HhaD48N showed that the “H-NS-blind” Hha mutant binds with higher affinity to StpA than the WT Hha protein. This result suggests that the interaction of Hha with StpA involves different residues in Hha from the ones described for the H-NS/Hha complex. Although the high homology exhibited between H-NS and StpA, under some circumstances both proteins might have distinct biological functions (Deighan et al., 2000) and DNA-binding mechanisms (Lim et al., 2012). In this regard, it is tempting to speculate that several models of interaction of Hha with different members of the H-NS family might take place. Further structural studies will be required to resolve the Hha/StpA complex.

The use of the “H-NS-blind” Hha mutant permitted us to identify a group of genes regulated by Hha in an H-NS/StpA-independent manner. For this purpose, we compared the transcriptomic profiles of four different strains: WT, Δ*hha*, *hha*D48N, and Δ*stpA*. The results obtained drove us to the identification of a set of 120 genes regulated by Hha in an H-NS/StpA-independent manner. Among these 120 genes, 39 genes have been previously described to be regulated by H-NS and/or StpA (Supplementary Table S2; Baños et al., 2009; Lucchini et al., 2009). Nevertheless, this H-NS/StpA-dependent regulation does not necessarily imply protein–protein interactions with Hha. The 120 genes identified were grouped into the following functional categories: genes of unknown functions, pathogenicity islands, cell envelope, protein synthesis, and transport and binding of proteins. Several phenotypic studies were assayed to corroborate the transcriptomic data.

Since genes involved in iron uptake, *fepG*, *fepD*, *fepB*, and *entS*, were down-regulated in an *hha* mutant, we decided to check the siderophore content in the culture supernatant of different strains. The siderophore content was slightly lower in the Δ*hha* mutant compared to WT, *hha*D48N and Δ*stpA* strains, indicating that Hha could play a role in the expression of these genes. The down-regulation of *entS*, encoding the main enterobactin exporter protein in *Salmonella* and *E. coli* (Furrer et al., 2002; Methner et al., 2008) could be responsible for the faint difference of siderophores detected in the Δ*hha* mutant supernatant. Notably, secretion and transport of siderophores is a complex system, involving alternative transport systems. Although the detection of siderophores in the Δ*hha* mutant is lower than in any of the other strains under study, the mere presence of these iron-chelating compounds in Δ*hha* supernatants suggests the existence of another fully functional exporter system outside the Hha regulon. IroC, described as an enterobactin and salmochelin transporter (Hantke et al., 2003; Crouch et al., 2008), could replace EntS protein, therefore masking the decrease on the *entS* expression. Additionally, FepG, FepD and FepB proteins are associated with siderophore transport across the cytoplasmic membrane into the cytoplasm (Hantke et al., 2003; Zhu et al., 2005; Crouch et al., 2008). The down-regulated expression of these genes in an Δ*hha* mutant reveals that Hha, in addition to partially affect the siderophore exporter system, regulate the uptake of siderophores. However, siderophores that remain outside may be recognized by outer membrane receptors, such as IroN or FepA (Hantke et al., 2003) and stay in the periplasm waiting to be transported into the cytoplasm.

The modulatory role of Hha in the expression of the genes *flgN*, *motB*, *tcp*, and *tsr*, related to flagellar motility and chemotaxis, was assessed. The differences in motility exhibited by the strains used in the assay allowed us to discriminate between the regulation that could be held by Hha and different complexes (Hha/StpA and Hha/H-NS). The double mutant SV5015DY displayed a motile phenotype, which could be associated with the regulatory effects that can be played by Hha (in an H-NS independent-manner) and through its interaction with StpA. The existence of a modulatory function of Hha independently of H-NS and StpA in the expression of genes involved in motility was confirmed by using the SV5015DYS triple mutant. Complementation in *trans* of the *hha* mutation with the WT Hha or the “H-NS-blind” Hha mutant confirmed our hypothesis. Surprisingly, the *ydgT* mutation appears to cause an increase in motility compared to the WT strain. This result disagrees with previous studies that have reported a motile phenotype for the Δ*ydgT* mutant similar to the one exhibited by the WT strain (Wallar et al., 2011). However, differences in the composition of the motility plates could result in different swimming phenotypes. More studies will be required to understand the regulatory role of the YdgT protein in motility.

Transmission electron microscopy allowed us to determine that the non-motile SV5015HY and SV5015HYS mutant strains presented flagella on their surface, although the number of flagella was slightly lower than in strains SV5015DY and SV5015DYS. Among the Hha-dependent genes (H-NS/StpA-independent), *motB* was found to be down-regulated. This gene encodes a flagellar motor component that, with MotA, form the proton-channel complex of the proton-driven bacterial flagellar motor (Morimoto et al., 2010; Morimoto and Minamino, 2014). Previous studies determined that the non-motile phenotype observed in an Δ*hha ΔydgT* mutant is due to a decrease in the expression of the genes encoding the transcriptional regulator FlhD_4_C_2_, mediated by PefI-SrgD. In other words, the expression of the PefI-SrgD repressor complex is under the negative control of Hha and YdgT. Wallar et al. (2011) showed that, in an *Δhha ΔydgT* double mutant, low levels of FlhD_4_C_2_ are translated in the down-regulation of flagellar promoters and this prompts the loss of surface flagella and motility. It is worth noting that in this work we have visualized cells grown on motility plates, whereas in the above mentioned work cells were grown in LB medium. Such differences in the methodology might explain the differences observed in the phenotype of the double mutant. Taking together, our data suggest that the loss of motility in SV5015HY and SV5015HYS strains could be explained by both: (i) the role accomplished by Hha and YdgT, through its interaction with H-NS, on the negative regulation of PefI-SrgD; (ii) the regulatory role that Hha, independently of H-NS, plays on flagella at the motor level.

The transcriptomic data obtained in this work suggest a role for the Hha protein in the regulation of expression of the *ssrA* and *ssrB* genes involved in SPI-2 regulation. Transcription assays in LB medium using the *ssrA::lacZ* transcriptional fusion allowed us to demonstrate that the *hha* mutant exhibits a higher expression of the *ssrA* gene compared to the wild type and the *hha*D48N mutant, suggesting a regulatory role of the Hha protein independently of H-NS. Moreover, this effect is exalted when the Δ*hha*Δ*ydgT* and *hha*D48N Δ*ydgT* genetic backgrounds are compared. In contrast, when the expression of *ssrA* was tested in cells grown under SPI-2-inducing conditions, LPM medium, no differences were observed between the Δ*hha* and *hha*D48N genetic backgrounds. This behavior is in good harmony with the survival and replication within macrophages assays. The results suggest that the regulation of SPI-2 genes in intracellular conditions is dependent on the complex Hha/H-NS, since the presence of a protein “H-NS blind” does not hold a modulatory effect, whereas a clear Hha repressor function of SPI-2 genes is observed in extracellular conditions. The role of Hha in SPI-2 genes expression has been previously reported although it is likely that H-NS is also involved in this regulation (Silphaduang et al., 2007). However, in this work we describe a role for Hha on SPI-2 regulation independently of its interaction with H-NS. Altogether these data highlight the role of the Hha protein as a repressor of SPI-2 genes when the cells are outside the intracellular environment. Furthermore, the results confirm that the Hha/H-NS regulatory complex is essential for the proper regulation of *ssrAB* during the intracellular phase of infection of *S*. Typhimurium (Coombes et al., 2005; Silphaduang et al., 2007).

In this work, we have described for the first time an Hha regulon independent of H-NS. It is tempting to speculate that, under specific environmental conditions, Hha, H-NS, and StpA could play different regulatory roles, acting as complexes or separately. By this means, regulation held by Hha, Hha/H-NS, and Hha/StpA guarantees the fine-tuning of the different regulons. Some of the genes identified in this work are good candidates for this dual regulation under different environmental conditions, as exemplified by the SPI-2 regulators SsrAB, as well as previously suggested for the regulation of *hlyABCD* or *hilA* gene expression (Nieto et al., 2000; Boddicker and Jones, 2004; Queiroz et al., 2011). In light of the above, the differential expression of *hns* and *hha* is likely to influence the abundance of the different complexes in the cell at any time. The expression of *hns* is relatively constant along the growth phase (Schröder and Wagner, 2002) and at different temperatures (Göransson et al., 1990), whereas *hha* expression is enhanced in LB medium at high temperature (37°C) and early stationary phase of growth (Paytubi et al., 2014). Therefore, Hha might become an important cellular protein under environmental conditions encountered by the bacterium during the infection process. Moreover, to accomplish the regulatory role described, we cannot rule out interactions of Hha with proteins other than H-NS or StpA.

The data obtained in this work indicates that 37.5% of the genes that belong to the Hha regulon correspond to HGT DNA (6.7% plasmid- and 30.8% chromosomally encoded genes). The percentage of HGT^2^ DNA in *Salmonella* LT2 is approximately 10%. It is hence remarkable that the Hha regulon in *Salmonella* is enriched with HGT sequences. A recent study demonstrated that, in *E. coli* K-12, Hha/YdgT function in complexes with H-NS/StpA to regulate expression of HGT genes (Ueda et al., 2013). Slight differences in the HGT content of both species, *E. coli* K-12 and *S.* Typhimurium, might account for the lack of identification of a specific Hha regulon in *E. coli*. Additionally, a significant proportion of the *Salmonella* “unique” genes are involved in virulence (Conner et al., 1998) where Hha plays a relevant role.

Future work will be aimed at precisely defining the molecular mechanism underlying the modulatory role of Hha protein.

## Conflict of Interest Statement

The authors declare that the research was conducted in the absence of any commercial or financial relationships that could be construed as a potential conflict of interest.

## Footnotes

1 http://bioinfo.ut.ee/primer3 (Rozen and Skaletsky, 2000).

2 http://genomes.urv.es/HGT-DB (Garcia-Vallve et al., 2003).
