# Supplementary material for: Hha has a defined regulatory role that is not dependent upon H-NS or StpA
Source: Front Microbiol. 2015 Jul 30;6:773. doi: 10.3389/fmicb.2015.00773 (PMC4519777; doi:10.3389/fmicb.2015.00773)
Supplement: Supplementary file 3 [file Data_Sheet_3.DOCX]

**Table S3**. Fold change expression of *motB*, *ssrA* and *sipB* of samples SV5015H, SV5015D, SV5015S, SV5015HY and SV5015DY compared to wild-type strain SV5015. Data means of three independent experiments and the standard deviation is shown.

| **Strain** | **Mean ± S.D.** | | |
| --- | --- | --- | --- |
|  | *motB* | *ssrA* | *sipB* |
| SV5015H | +1.12 ± 0.39 | +2.53 ± 0.45 | +2.53 ± 1.04 |
| SV5015D | +1.97 ± 0.57 | +3.75 ± 1.06 | +2.36 ± 0.27 |
| SV5015S | +4.15 ± 0.55 | +3.65 ± 1.56 | +4.86 ± 0.47 |
| SV5015HY | -3.69 ± 0.13 | +16.7 ± 5.12 | +47.20 ± 5.10 |
| SV5015DY | -1.82 ± 0.17 | +7.15 ± 1.57 | +15.98 ± 6.51 |
