# Supplementary figures and images for: Hha has a defined regulatory role that is not dependent upon H-NS or StpA
Source: Front Microbiol. 2015 Jul 30;6:773. doi: 10.3389/fmicb.2015.00773 (PMC4519777; doi:10.3389/fmicb.2015.00773)

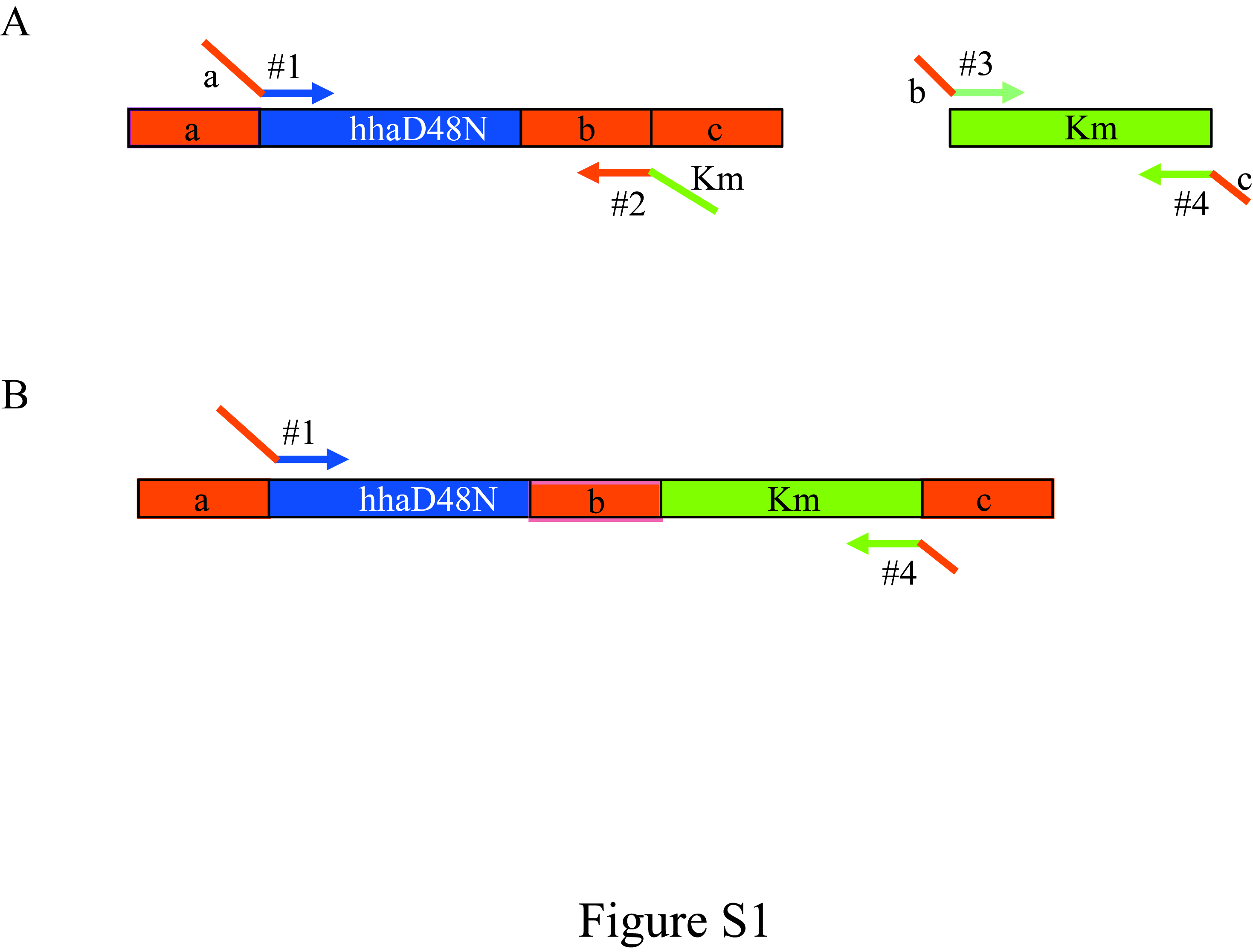

Supplement: Supplementary file 4 [file Image_1.TIF]

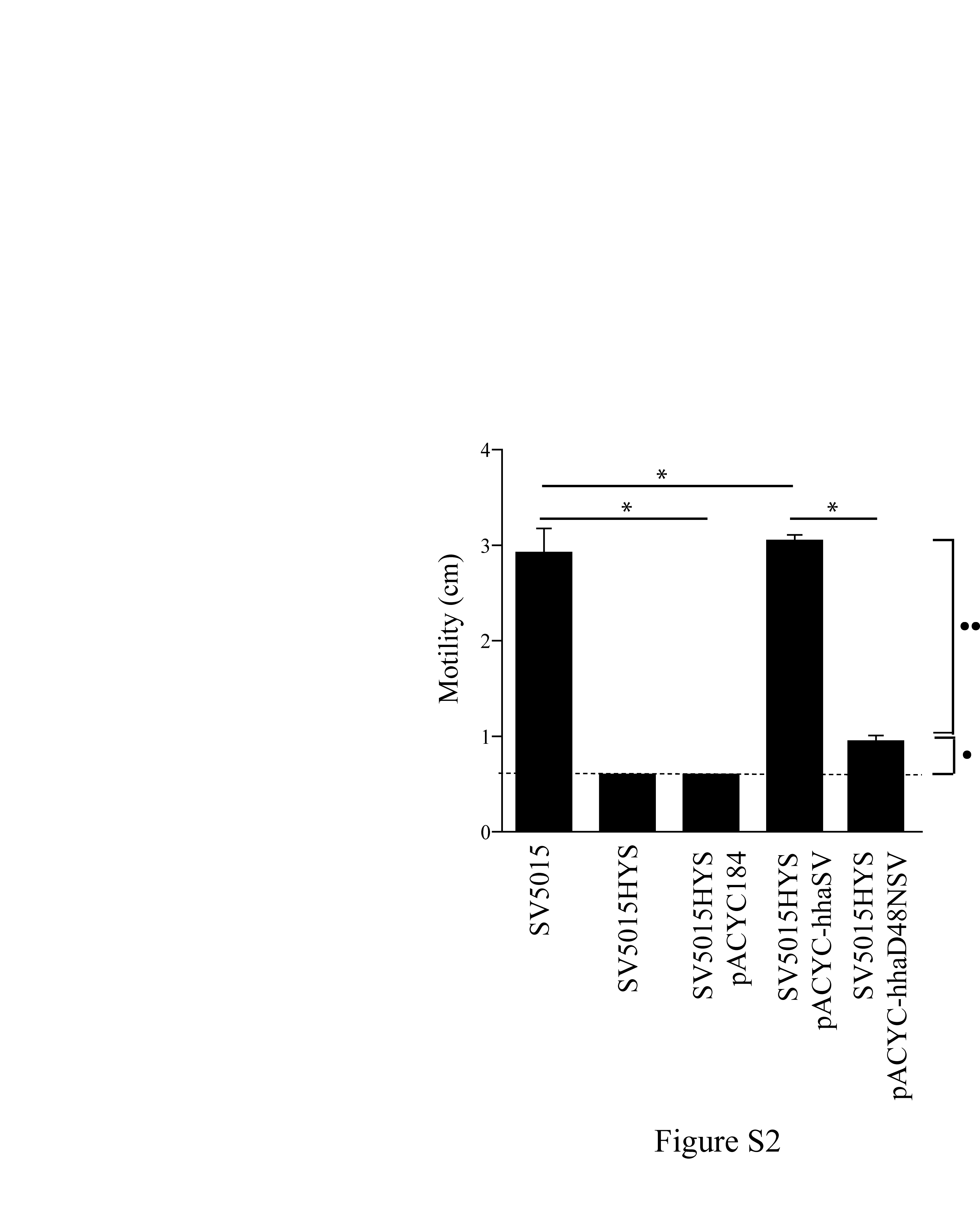

Supplement: Supplementary file 5 [file Image_2.TIF]
